# Francis Anstie

**Published:** 1874-11

**Authors:** 


Francis Anstie.—"It is impossible, in dwelling upon the rare qualities which characterized Dr. Anstie, to separate the personal, which endeared him to his intimate friends, from the intellectual, by which his name became familiar to the profession and the public. He was a physician of the type which is the pride of English physic and the crown of our schools of Medicine. Or rather it should be said he was a typical physician of this class. His intellectual culture was not inferior to his strictly medical culture, and this was of the highest. The one, in fact, formed, as it were, the fitting setting for the other. Complete in the technical knowledge of his profession, he brought to its application in actual life that happy combination of scientic and practical acumen, conjoined with a peculiar charm of manner, which makes the physician in the truest and best sense of the word. Prompted by a thorough love of medicine, he labored closely and hard at some of its most complicated problems; and he has left results of enduring value both scientific and practical. His scientific work is characterized by keen insight into the problems with which he dealt, and by a rare appreciation of the light in which his researches should be regarded, as contributing, on the one hand, to existing knowledge of the subjects to which they referred, and, on the other, to the furtherance of investigation. His practical work was distinguished by the skill with which he sought to educe principles of treatment from pathological and physiological data, and rationalized necessary empiricisms. Widely read in the literature of his profession, he brought a richly stored memory to his editorial and literary duties. He was an eager upholder of the dignity of medicine, and an unsparing critic of whatever militated against the good name of the profession within or without its ranks. A clear and incisive style gave power to a pen which, if it were keen, was never unkind, and which has performed an active and influential part in all the great questions which have agitated medicine in its social and political bearings for some years past.

"The substantial fruit's of Dr. Anstie's labors were about to be gathered when, in the earnest pursuit of a duty, he accidently received the infection which killed him. Medicine, although rich in sterling workers, can ill afford to lose such a man, and the manner of his death aggravates the painful sense of his loss. It was too costly a life to have been sacrificed prematurely in so pitiful a way."—*London Lancet.*

